# The clinical relevance of early identification and treatment of sleep disorders in mental health care: protocol of a randomized control trial

**DOI:** 10.1186/s12888-020-02737-3

**Published:** 2020-06-24

**Authors:** Fiona M. ter Heege, Teus Mijnster, Maaike M. van Veen, Gerdina H. M. Pijnenborg, Peter J. de Jong, Gretha J. Boersma, Marike Lancel

**Affiliations:** 1 GGZ Drenthe Mental Health Institute, 9404 LA Assen, The Netherlands; 2 Department of clinical and Developmental Neuropsychology, University of Groningen, Groningen, the Netherlands; 3 Department of Clinical Psychology and Experimental Psychopathology, University of Groningen, Groningen, the Netherlands

**Keywords:** Sleep disorders, Early detection, Early treatment, Mental disorders, Psychiatric outcome, Quality of life

## Abstract

**Background:**

Sleep disorders are a risk factor for developing a variety of mental disorders, have a negative impact on their remission rates and increase the risk of relapse. Early identification and treatment of sleep disorders is therefore of paramount importance. Unfortunately, in mental health care sleep disorders are often poorly recognized and specific treatment frequently occurs late or not at all. This protocol-paper presents a randomized controlled trial investigating the clinical relevance of early detection and treatment of sleep disorders in mental health care. The two aims of this project are 1) to determine the prevalence of sleep disorders in different mental disorders, and 2) to investigate the contribution of early identification and adequate treatment of sleep disorders in individuals with mental disorders to their sleep, mental disorder symptoms, general functioning, and quality of life.

**Methods:**

Patients newly referred to a Dutch mental health institute for psychiatric treatment will be screened for sleep disorders with the self-assessment Holland Sleep Disorders Questionnaire (HSDQ). Patients scoring above the cut-off criteria will be invited for additional diagnostic evaluation and, treatment of the respective sleep disorder. Participants will be randomly assigned to two groups: Immediate sleep diagnostics and intervention (TAU+SI-T0), or delayed start of sleep intervention (TAU+SI-T1; 6 months after inclusion). The effect of sleep treatment as add-on to treatment as usual (TAU) will be tested with regard to sleep disorder symptoms, general functioning, and quality of life (in collaboration with a psychiatric sleep centre).

**Discussion:**

This trial will examine the prevalence of different sleep disorders in a broad range of mental disorders, providing information on the co-occurrence of specific sleep and mental disorders. Further, this study is the first to investigate the impact of early treatment of sleep disorders on the outcome of many mental disorders. Moreover, standard sleep interventions will be tailored to specific mental disorders, to increase their efficacy. The results of this trial may contribute considerably to the improvement of mental health care.

**Trial registration:**

This clinical trial has been retrospectively registered in the Netherlands Trial Register (NL8389; https://www.trialregister.nl/trial/8389) on February 2th, 2020.

## Background

Sleep problems are among the most frequent complaints in individuals with a mental disorder. Therefore, sleep disorders are common in individuals with mental disorders. Chronic insomnia, for example, is estimated to affect more than 40% in schizophrenia spectrum disorders [1], compared to approximately 6% in the general population [2]. Furthermore, insomnia complaints were reported in more than 50% in individuals with an affective disorder [3]. Six categories of clinical sleep disorders are identified in the International Classification of Sleep disorders (ICSD-3); insomnia, sleep related breathing disorders (SRBD), central disorders of hypersomnolence (hypersomnia), circadian rhythm sleep-wake disorders (CRSD), parasomnias, and sleep-related movement disorders [4]. Although insomnia prevalence has been investigated in connection with several different mental disorders, prevalence rates for the other categories of sleep disorders have been less or not at all studied in connection to the presence of mental disorders. More detailed information on the prevalence of a certain sleep disorder in connection to particular mental disorders could improve treatment strategies and may even lead to the identification of novel etiological pathways underlying the respective sleep disorders.

Studies suggest that sleep disorders increase the risk for the development of mental disorders, and worsen treatment outcome [5–7]. For instance, insomnia has been shown to have prognostic validity for the development of posttraumatic stress disorder (PTSD) [6, 8] and depression [9, 10]. Moreover, the already high vulnerability for suicidal behavior in individuals with mental health problems is further increased in those with poor sleep [11, 12]. Thus, early and adequate treatment of sleep disorders in individuals with mental disorders may be of paramount importance, and might well lead to better mental health outcomes and improved quality of life.

Despite the widespread use of sleep medication in psychiatry, cognitive behavioral therapy for insomnia (CBT-I) is currently considered the first-choice treatment of chronic insomnia. CBT-I consists of psychoeducation, sleep hygiene, relaxation training, stimulus control therapy, sleep restriction therapy and cognitive therapy. CBT-I is safe and effective (reviewed in [13]), with superior long-term effects compared to sleep medication (in [14]). In individuals with both insomnia and depression, CBT-I was found to both alleviate insomnia complaints and reduce depression severity [15–18]. Furthermore, in individuals suffering from posttraumatic nightmares, the treatment of nightmares with imagery rehearsal therapy (IRT) showed promising results in reducing both insomnia and nightmare frequency, as well as other global PTSD symptoms [19]. Although these standard sleep disorder treatments are successful in the general population, within individuals with mental disorders problems with compliance may arise, for example because the commonly used procedures may elicit distress that interferes with the efficacy of treatment. Therefore, treatment protocols used in this study were adapted to better fit the targeted population.

Despite a high occurrence of sleep disorders and established negative effects on mental health, little attention is paid to sleep problems in mental health care. Sleep disorders are frequently diagnosed years after onset; years in which poor sleep already exerted detrimental effects on physical and mental health, daytime functioning and quality of life. Clearly, a lot can be gained in mental health care by a timely delivery of sleep disorder treatment. This paper presents the protocol of a randomized controlled trial, in which the prevalence of distinct sleep disorders will be assessed and the clinical relevance of early sleep disorder treatment in mental health care will be tested.

### Aims and objectives

The overall aim of this study is to investigate the clinical relevance of early identification and adequate treatment of sleep disorders that co-occur with mental disorders.

The primary objectives of the study are to investigate:

1) The prevalence of the different six categories of sleep disorders (insomnia, SRBD, hypersomnia, CRSD, parasomnias, and sleep-related movement disorders) in connection to specific mental disorders.

2) Whether screening for and early treatment of sleep disorders exerts positive effects on sleep, treatment outcomes (symptom severity/remission of mental disorder), and quality of life.

Sex differences in both sleep and mental disorders are common; therefore, the effect of sleep disorder treatment may differ between the sexes. Based on this, a secondary objective of this study is to investigate whether there is an interaction effect between sex and treatment of sleep disorders on sleep, mental disorders symptoms (symptom severity/remission of mental disorder), and quality of life.

## Methods

### Design

This study is designed as a randomized controlled trial in order to test the effects of early interventions for sleep disorders on both sleep problems and mental health outcome. Participants will be asked to complete several questionnaires concerning sleep, quality of life, general functioning and specific mental disorder symptomatology, at three different time points over the course of twelve months. Researchers and study participants are not blinded to the conditions of the study.

### Study population

All treatment seeking individuals who are newly registered at GGZ Drenthe Mental Health Institute (GGZ Drenthe, Assen, NL; https://ggzdrenthe.nl/) will be asked to participate in this study by their treating therapist. The population of this mental health institute consists of individuals diagnosed with a variety of mental health disorders. Most individuals are diagnosed with chronic and/or complex mental disorders and often present with co-morbid disorders. Based on the participants’ main diagnosis according to the *Diagnostic and Statistical Manual of Mental disorders -* Fifth edition (DSM–5) [20], participants will be subdivided into the following diagnostic groups: depressive disorder, bipolar disorder, anxiety disorder, PTSD, attention deficit (hyperactivity) disorder (AD(H)D), autism spectrum disorder (ASD), schizophrenia spectrum disorder (SSD), or personality disorder. Patients under the age of 18, unable to adequately read or speak Dutch, and/or deemed unfit (by their therapist) to fill out questionnaires will be excluded from the study.

### Procedure

As part of the intake procedure of the mental health institute, routine outcome assessment (ROA) measurements are conducted in most departments of the institute, using an established online platform [21]. We will use this infrastructure, to reduce the burden to the participants and ensure data quality. Participants will be asked to complete questionnaires prior to the intake procedure (T0), 6 (T1), and 12 (T2) months later.

To screen for sleep disorders, patients fill out the Holland Sleep Disorders Questionnaire (HSDQ) [22, 23]. Patients who score above the cut-off score for any specific category of sleep disorders will be invited to participate in this study. After being informed about the study procedures, they will be asked for informed consent.

**Fig. 1:**
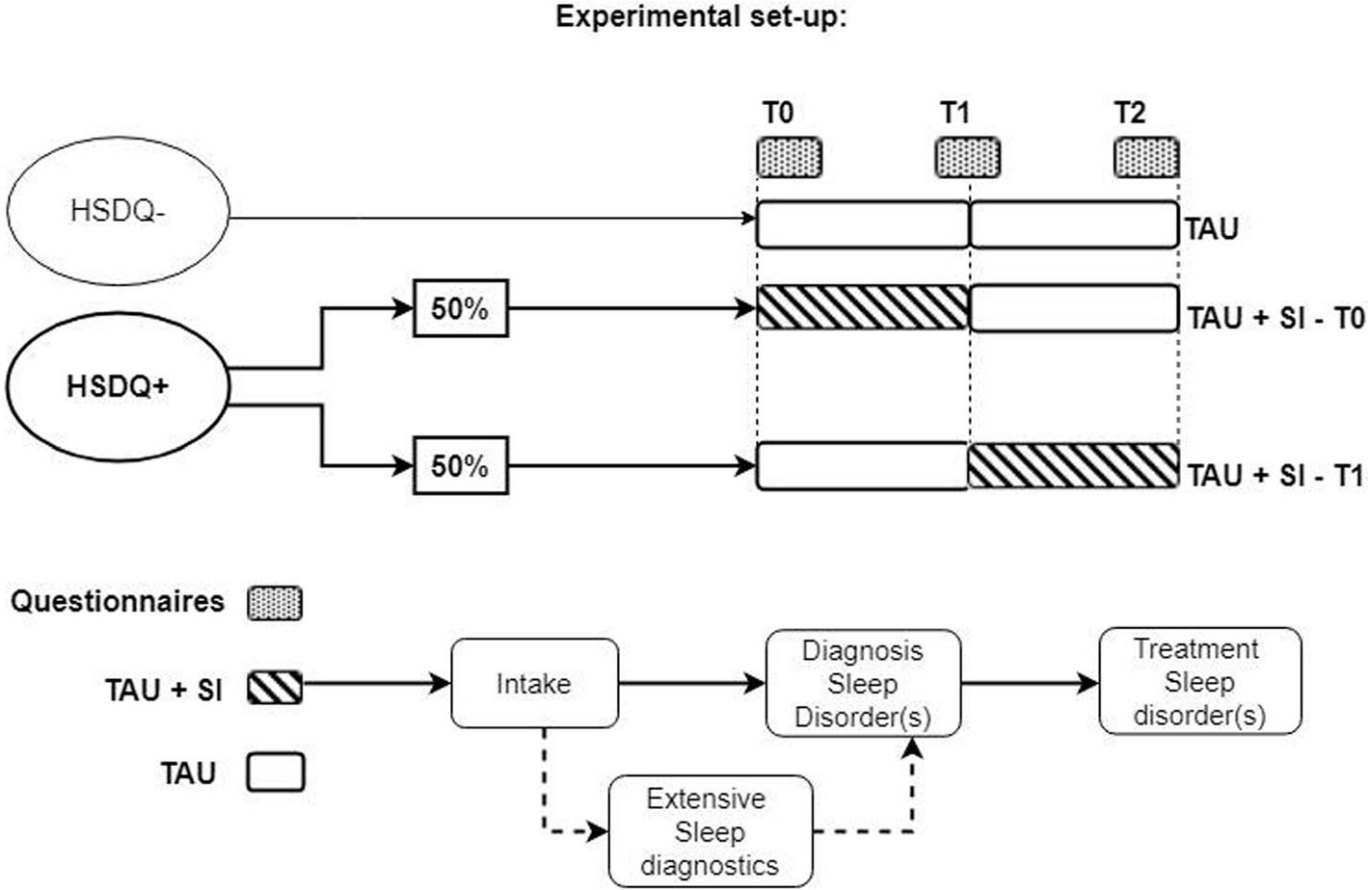
Study procedure. Patients who score above the cut-off score for any type of sleep disorder on the Holland Sleep disorders Questionnaire (HSDQ+) will be invited for extensive diagnostic evaluation, followed by patient-targeted sleep treatment. Those participants will be randomly divided in one of two conditions: in TAU+SI-T0 sleep diagnosis and sleep intervention (SI) will start immediately (T0), in addition to the treatment as usual (TAU). In TAU+SI-T1 sleep diagnosis and SI will start after 6 months (T1). Questionnaires will be filled in at T0, T1 and T2 (after 12 months), measuring sleep, general functioning, specific mental disorder symptoms, and quality of life. For patients scoring below the cut-off score for a sleep disorder (HSDQ-), participation is limited to filling out the questionnaires at T0

For both the experimental and control conditions, participants will continue their treatment as usual (TAU) throughout the study. After stratification for type of mental disorder, participants will be randomly assigned (using Sealed Envelope software) to either the experimental or control condition. In the experimental condition sleep intervention(s) (SI) will start immediately at T0 (TAU+SI-T0). In the control condition, the sleep intervention(s) will first start six months after inclusion, at T1 (TAU+SI-T1). This allows to test the effects of early interventions for sleep disorders on mental disorder symptoms, general functioning, sleep, and quality of life. Sleep interventions generally start with extensive diagnostic evaluation, followed by patient-targeted sleep treatment. For individuals scoring below the cut-off score for a sleep disorder on the HSDQ, participation is limited to filling out the questionnaires at T0. An exception to randomised allocation will be made for participants in the control condition whose HSDQ result or intake interview indicates a very severe sleep disorder, such as Narcolepsy. These participants will be immediately referred to a sleep centre, regardless of original randomization. These patients will be included in the prevalence estimate, but excluded from the analysis of the treatment effects. An overview is provided in Fig. 1.

This research project will be conducted in collaboration with a sleep centre for psychiatry. Anamnestic interviews, diagnostic assessments and sleep interventions will be provided by trained staff.

### Study outcomes

Primary outcomes are 1) the prevalence of different sleep disorders as a function of individuals’ mental disorders (depressive disorder, bipolar disorder, anxiety disorder, PTSD, AD(H)D, ASD, SSD or personality disorder), and 2) the effect of sleep interventions on sleep, severity of specific symptoms of mental disorders, general functioning, and quality of life. Secondary outcomes are 1) the effects of sleep interventions on objective outcome parameters, for example use of sleep/sedative medication, number of hospitalizations and length of stay and, 2) differences between genders in the prevalence of specific sleep disorders and sleep intervention effects.

First, we expect that the prevalence of most sleep disorders among individuals with mental disorders will exceed those in the general population. Second, concerning the adjusted sleep interventions, we expect that the treatment of sleep disorders, tailored to mental disorders, will improve sleep, recovery from mental disorder symptoms, and quality of life in most patients. We hypothesize that the effect of added sleep intervention(s) will be larger than the effect of TAU alone in the TAU-SI-T1 group.

### Measures/ materials

All scales used in this study will be administered in the Dutch language and have been validated for use in Dutch.

#### Sleep

The HSDQ [22] is a 32-item self-assessment questionnaire for sleep disorders, based on the International Classification of Sleep disorders (ICSD-2) [24]. Each item consists of a self-description (i.e. “I have difficulty falling asleep”) and participants are instructed to respond on a 5-point Likert scale indicating if the stated item applies to them for the past 3 months (1= “not at all”, 2 = “usually not”, 3 = “sometimes”, 4 = “usually”, 5 = “completely”). This questionnaire is validated (Cronbach’s alpha (α) = .90) [22] and screens for the following categories of sleep disorders: Chronic insomnia (α = .92), parasomnia (α = .85), CRSD (α = .84), hypersomnia (α = .78), sleep-related movement disorders (i.e. restless legs syndrome (RLS)) (α = .81), and SRBD (i.e. obstructive sleep apnea (OSA)(α = .62)) [23]. The total score of the HSDQ distinguishes individuals with a clinically diagnosed sleep disorder from individuals without sleep complaints (area under the curve, *P*(A) = .95) and the diagnostic accuracy of the HSDQ is high (kappa = .80) [22]. In the present study, the HSDQ will be used for screening purposes and to assess sleep disorder prevalences. Mean scores will be calculated and the following cut-off criteria will be used: Sleep disorder > 2.02; chronic insomnia > 3.68; parasomnia > 2.42; CRSD > 3.42; hypersomnia > 2.9; sleep-related movement disorder > 2.7; SRBD > 2.87 [22]. For the analysis of sleep treatment effect on response of the clinical sleep disorder(s), the HSDQ total and subscale scores will be used as outcome variables.

#### Quality of life

##### *Manchester Short Assessment of Quality of Life* (MANSA)

The MANSA will be used to assess quality of life. The MANSA is a 16-item self-rating scale with sufficient internal consistency (α = .74) [25]. The questionnaire addresses the following aspects: Living circumstances, work circumstances, financial circumstances, friendships, family relations, leisure time, personal safety, sexual relationships, mental health and physical health. There are 4 questions that can be answered with “yes” or “no”, the other items will be scored on a 7-points scale: “could not be worse”, “dissatisfied”, “mostly dissatisfied”, “neutral”, “mostly satisfied”, “satisfied” or “could not be better”. A total score will be calculated whereby a higher score indicates a higher quality of life.

##### *Individual Recovery Outcome Counter* (I-ROC)

In this study we will use the I-ROC, a 12-item self-rating scale designed to measure personal recovery. The I-ROC is a valid and reliable recovery outcomes measure, with a sufficient high internal consistency (α = .86) [26]. The questionnaire focusses on the following themes: Mental health, life skills, safety and comfort, physical health, exercise and activity, purpose and direction, personal network, social network, valuing myself, participation and control, self-management, and hope for future [26]. Participants will be asked to score each item on a 6-point Likert scale, ranging from 1 (= “never”) to 6 (= “all of the time”), where each rating refers to the past 3-month period. A higher total score intents to represent a greater level of personal recovery.

#### General functioning

To assess changes in general functioning, the Outcome Questionnaire (OQ-45) [27] will be employed. In this 45-item self-report questionnaire participants will be asked to answer the items based on how they have felt over the past week on a 5-point Likert scale, ranging from 0 (= “never”) to 4 (= “always”). The OQ-45 is a validated instrument with good internal consistency (α = .93) [27]. The OQ-45 was shown to be sensitive to treatment effects in heterogeneous patient samples [28]. The questionnaire originally consists of 3 different subscales: 1) Subjective Distress, 2) Interpersonal Relations and 3) Social Role. In this study we will use the Dutch version of the OQ-45, in which an extra subscale has been added and validated: Anxiety and Somatic Distress [29]. The psychometric properties of the Dutch OQ-45 are adequate and similar to the original instrument [29]. The total score and the four subscale scores of the OQ-45 will be used as outcome parameters.

#### Specific mental disorder symptoms

One (or more) of the following questionnaires, depending on the participant’s main DSM-5 diagnosis, will be used to assess specific symptomatology. Total scores will be used as outcome parameters and participants will be invited to fill out the questionnaire at all three time points.

##### Quick inventory of depressive symptomatology, self-report (QIDS-SR_16_)

The QIDS-SR_16_ [30] is a 16-item self-report scale that will be used to assess depressive symptomatology, in individuals with a depressive or bipolar disorder diagnosis. Participants are asked to answer the items by selecting one out of four statements that fitted them best in the last week. The QIDS-SR_16_ is a validated instrument with good internal consistency (α = .86) and sensitivity to symptomatic change following treatment [30].

##### Altman self-rating mania scale (ASRM)

The ASRM [31] is a 5-item self-rating questionnaire that measures mania symptoms. This questionnaire is used in individuals with a bipolar disorder diagnosis, in addition to the QIDS. In each item participants will be asked to select one out of five statements that best summarizes the way they felt in the last week. The ASRM is a validated scale, with sensitivity to change following treatment [31].

##### Spielberger state-trait anxiety inventory, Dutch-Y (STAI-DY)

The STAI is a brief self-report scale designed to measure and differentiate between anxiety as a trait and a state. In this study a Dutch version (STAI-DY) [32] will be used to assess anxiety symptoms in individuals with anxiety disorder diagnosis. Participants are asked to only fill out the state section of the STAI-DY. They will be asked to rate 20 statements on a 4-point scale ranging from “not at all” to “very much so”. The STAI-DY has been validated and the internal consistency is good (α between .91 and .95) for both individuals who are treated in outpatient and inpatient centers for mental health care [32].

##### Posttraumatic stress disorder checklist for DSM-5 (PCL-5)

The PCL-5 [33] is a 20-item self-respond measure of PTSD symptomatology, in individuals with a PTSD diagnosis. Participants are asked to describe their worst stressful experience and after this, to provide a symptom severity rating that ranges from (0=) “not at all” to (4=) “extremely”. The PCL-5 is a psychometrically sound instrument with good internal consistency (α = .94) [34] and useful for detecting clinical change over time in symptoms after treatment [35].

##### Adult ADHD self-report scale (ASRS-v1.1)

In this questionnaire participants are asked to rate a list of symptoms on a 5-point scale, ranging from (0=) “never” to (4=) “very often”, in order to assess ADHD symptoms in individuals with an ADHD diagnosis. The ASRS originally consists of 18 items. In this study we will make use of a shorter form of the ASRS (ASRS-v1.1), in order to reduce the burden to the participants. This 6-item screening version has shown to outperform the full version in sensitivity (68.7% versus 56.3%) and specificity (99.5% versus 98.3%) [36].

##### Autism spectrum quotient (AQ-50)

The AQ [37] will be used to assess autism-related symptoms in individuals with an ASD diagnosis. The AQ consists of 50 items assessing personal preferences and habits. Participants rate to what extent they agree or disagree with the listed statements on a 4-point Likert scale with answer categories: “definitely agree”, “slightly agree”, “slightly disagree”, and “definitely disagree”. The AQ is a valid and reliable instrument to assess individual differences in autistic traits and the internal consistency of the Dutch AQ is satisfactory (α = .71) [38].

##### PHAMOUS-basis psychosevragen

This short questionnaire will be used to assess symptom severity in individuals with a SSD diagnosis. Participants are asked to indicate if they have experienced the nine listed symptoms in the past 14 days by answering: 0 = “no”, 1 = “yes, probably”, or 2 = “yes”. The average item-score will be used as outcome variable. This questionnaire is currently being validated.

##### Positive and negative syndrome scale (PANSS)

In addition to the “PHAMOUS-basis psychosevragen”, in individuals with a SSD diagnosis assessments will be made on positive and negative symptoms at T0 and T2 as part of the ROM, using the Positive and Negative Syndrome Scale (PANSS) [39] interview. The PANSS is a validated 7-point, 30-item rating instrument, with each point representing increasing levels of psychopathology: 1 = “absent”, 2 = “minimal”, 3 = “mild”, 4 = “moderate”, 5 = “moderate-severe”, 6 = “severe”, and 7 = “extreme” [39].

### Sleep disorder diagnosis

The sleep diagnostic process depends on the suspected sleep disorder(s) indicated by the HSDQ. Each participant scoring positive for one or more (types of) sleep disorder(s) will be invited for a sleep diagnostic interview by trained psychologists. Participants will also be asked to complete one or more short questionnaires (5–10 min) during this interview, depending on the HSDQ-indicated sleep disorder(s). The following questionnaires will be used: the *Pittsburgh Sleep Quality Index* (PSQI) [40] for subjective quality of sleep; *Insomnia Severity Index* (ISI) [41] in participants with HSDQ-indicated Insomnia disorder; *Morningness - Eveningness Questionnaire* [42] in participants with HSDQ-indicated CRSD; *Epworth Sleepiness Scale* (ESS) [43] in participants with HSDQ-indicated SRBD and/or hypersomnia and; *International RLS study group rating scale* (IRLS) [44] in patients with HSDQ-indicated RLS. An additional file is added, and describes the sleep-oriented questionnaires in more detail (see Additional file 1). In order to evaluate the effect of sleep disorder treatment, the same questionnaires will also be filled out by the participants directly following the sleep intervention.

The diagnostic interview takes around 30–60 min. Hereafter, participants will be asked to wear an actigraph (Motionwatch 8, CamNtech) for two weeks in case of CRSD, and for one week in all other cases. This actigraph is a diagnostic instrument in the form of a wristband that registers bodily activity in order to asses objective sleep quality and sleep-wake pattern. Additionally, participants will be asked to complete a sleep diary for the same time period they wear the actigraph, in which they note their bedtimes, estimated sleep latency, total sleep duration, sleep quality, and number of awakenings. If further information is needed for diagnosis, additional diagnostic measurements will be made, such as polysomnography, Dim Light Melatonin Onset (DLMO) measurement to determine the circadian rhythm in CRSD and Multiple Sleep Latency Test (MSLT) to evaluate hypersomnia. For diagnosing sleep disorders, ICSD-3 criteria [4] will be followed. Final diagnosis will be made during a multidisciplinary consultation with a somnologist, a somno-technologist and psychologists. Complex cases will additionally be discussed with a neurologist, pulmonary-specialist, and/or psychiatrist.

### Sleep disorder treatment

Based on the sleep diagnosis, a sleep treatment plan will be made by the multidisciplinary team described above. Furthermore, the treatment plan will be discussed with the participant’s mental health professional to ensure it fits with the patient’s overall treatment plan. Examples of standard sleep interventions are: psychotherapies, (e.g. CBT-I and IRT), chronotherapy (e.g. light and dark therapy), treatment for sleep related breathing disorders (e.g. continuous positive airway pressure (CPAP)) and/or medication)(overview provided in Table 1). All sleep disorder treatments are well established and follow international guidelines. Also, current treatments were evaluated by patient representatives from each mental disorder diagnostic group and by experienced practitioners of the sleep centre, prior to the start of the study. Where appropriate, treatments were adjusted to decrease the burden on the participants, to increase treatment compliance, and/or efficacy. Treatments will be delivered by trained psychologists and in case of medication use and/or advice a psychiatrist will be involved in the treatment as well.

**Table 1:** *Overview of indicated sleep interventions*

Sleep disorder	Treatment options
**Insomnia**	CBT-I; lifestyle advise; adjustments of medication, treatment with medication
**OSAS**	Lifestyle advise; CPAP; positional trainer; mandibular repositioner appliance (MRA); adjustments of medication
**Delayed Sleep Phase Syndrome (DSPS)**	Lifestyle advise; chronotherapy; treatment with melatonin
**RLS/PLMD**	Lifestyle advise; adjustments of medication; treatment with medication
**Hypersomnia**	Daily scheduled nap; lifestyle advise; treatment with medication
**Nightmare disorder**	IRT; Eye Movement Desensitization and Reprocessing (EMDR); treatment with medication; medication advise

### Sample size calculations

Power analysis were performed using G*Power software [45]. Based on previous studies [18, 46], we aim at an effect size of 0.4 (Cohen’s d) for the outcome variables “quality of life”, “general functioning” and “mental disorder specific symptoms”. Power analysis based with an α of .05 and a power of .8, resulted in a required group size of 48 patients per diagnostic group (24 TAU+SI-T0 and 24 TAU+SI-T1). Due to limitations in recruitment capacity in less prevalent disorders, differences across both sexes will only be examined in the relatively high prevalent disorders: depression and PTSS. Power analysis with a 2 × 2 experimental group design (TAU+SI-T0 versus TAU+SI-T1; and males versus females) as between subject factors and the three time points as within subject factors suggests that with an α of .05 and a power of .8 a group size of 92 per group (46 TAU+SI-T0 and 46 TAU+SI-T1) would be sufficient. This analysis assumes an equal sex distribution. Overall, a total of 472 participants will be included in this study (overview provided in Table 2).

**Table 2:** *Overview of targeted sample-sizes per diagnostic category*

Group	Prevalence (%)	Sec. analysis: yes/no	Required number per experimental group	Total required number per diagnostic subgroup	% of total population needed to include
**Depression**	17	Yes	46	92	15.0
**Bipolar disorder**	3	No	24	48	44.4
**Anxiety disorder**	10	No	24	48	13.3
**PTSD**	15	Yes	46	92	18.1
**ADHD/ADD**	9	No	24	48	14.7
**ASD**	4	No	24	48	33.1
**SSD**	5	No	24	48	26.7
**Personality disorder**	6	No	24	48	22.3
**Total**			**236**	**472**	

### Statistical analyses

#### Data exploration/ missing data

Separate analyses will be performed for the different questionnaires, using SPSS (version 23) and R software. If data are not normally distributed, data transformations will be performed. A data management plan was generated using online software and can be found at DMPonline (https://dmponline.dcc.ac.uk/).

If a participant stops participation halfway the study or if a specific questionnaire is missing at a specific time point this will be dealt with as follows: if the last data point (T2) is missing, the participant will be included in the analysis for the prevalence and in any analysis focussed on de comparison between T0 and T1. For those participants that are missing a complete questionnaire at T1, but do have the T2 measurements, T1 will be treated as a missing data point and imputed using multiple imputation. If a participant is missing both the T1 and T2 data point, this participant will only be included in the prevalence analysis.

Because we make use of a delayed start for the sleep treatment in the TAU-SI-T1 group, there is a possibility that some participants in the TAU-SI-T1 control group score above the cut-off score for a sleep disorder on the HSDQ at T0, but below the cut-off score for a sleep disorder at T1. In this case, these participants will be informed about the change in their HSDQ score, and that they are no longer eligible for sleep disorder treatment, and thus excluded from the treatment part of the study. Another scenario is that a participant scores above a cut-off score of the HSDQ, but that the sleep disorder is not confirmed in the diagnostic evaluation. In this case, the participant will also be excluded from the treatment part of the study.

#### Prevalence

Descriptive statistics will be used to assess the prevalence of the different sleep disorders over the entire study population and within different subgroups of mental disorders. The prevalence of the six different types of sleep disorders (based on the HSDQ indicated diagnosis) will be calculated as a frequency for the total study population and for the eight mental disorder diagnostic groups separately. Differences in prevalence between the various mental disorder diagnostic groups will be computed using chi-square and/or Phi or Cramer-V analysis. The Standardized incidence ratio (SIR) will be calculated for the clinical population relative to reported prevalence of the different sleep disorders in the general population.

#### Effect of sleep disorder treatment

Nearly all sleep disorders induce disturbed sleep and consequently result in a sleep deficit. We hypothesize that the sleep deficit, rather than the underlying disorder, may be most influential on the added burden of the sleep disorder on the patient’s mental well-being. Therefore, in the analysis of the effects of added sleep interventions, no distinction will be made between the different all types sleep disorders and all treatments will be lumped together. The effects of added sleep intervention in all participants with a sleep disorder will be assessed at six and twelve months by comparison of treatment (TAU-SI-T0 and TAU-SI-T1) groups over time (T0, T1, and T2). Secondary analysis will focus on the interaction between treatment effect and sex differences. For statistical evaluation linear mixed models, separate for all outcome parameters, will be employed. A stepwise approach will be followed in which first results for crude models will be taken, after which models including potential confounders such as age, sex, and social demographic parameters are run.

These analyses will be performed in the total population for the quality of life and general symptom outcome parameters, and in the eight mental disorder diagnostic subpopulations (depressive disorder, bipolar disorder, anxiety disorder, PTSD, AD(H)D, ASD, SSD and personality disorder) for the specific mental disorder symptom parameters. Due to power limitations, secondary analysis will only be performed within the more prevalent disorders: depression and PTSD.

## Discussion

The overarching objective of this project is to develop and evaluate a procedure for early detection and treatment of sleep disorders in individuals with mental disorders in order to augment therapeutic outcome and enhance well-being.

The first aim of this study is to gain insight into the prevalence of the different sleep disorders as a function of specific mental disorders. If we would observe a high prevalence of a certain sleep disorder in connection to a particular mental disorder, extra attention can be paid and extra resources could be allocated to the detection and treatment of that specific sleep disorder within individuals who are diagnosed with this particular mental disorder. Additionally, the observation of a high prevalence of a certain sleep disorder in individuals with a specific mental disorder profile could lead to the identification of novel etiological pathways underlying the respective sleep disorders. This may improve mental health care and provide novel therapeutic targets.

The second aim of this project is to test whether sleep disorder treatment can improve the outcome of common treatment for mental disorders, as well as individual’s quality of life. For some mental disorders, treatment of a co-occurring sleep disorder might significantly improve mental well-being, whereas for other disorders it might have less incremental effects. That treatment of sleep disorders can improve mental health of individuals with mental disorders became evident in studies investigating treatment of insomnia in individuals with depressive disorder [15–18]. However, for other mental disorders and other sleep disorders, the importance of sleep disorder treatment for treatment outcome has only rarely been investigated. Thus, the current project will provide important complementary data allowing evaluation of the clinical relevance of treating sleep disorders in the context of distinct mental disorders.

This is one of the first studies to test the prediction that early identification and treatment of sleep disorders in addition to treatment as usual for mental disorders will result in superior treatment outcome. As an important strength, this study will include a large population with a wide variability in mental disorders and comorbid sleep disorders. Another asset of this project is that standard interventions for sleep disorders were adapted to vulnerable psychiatric populations, which will increase compliance and treatment efficacy. We anticipate that this study will contribute to both patient care as well as to our scientific understanding of the complex interaction between mental disorders and sleep disorders.

The goal of this project is to develop an effective paradigm for the early detection and treatment of comorbid sleep disorders for mental health care, in order to improve therapeutic outcome and patients’ well-being. To optimize the impact of such additional intervention and to prevent a detrimental impact of prolonged untreated sleep disorders on the course of individuals’ mental disorders, the current project focusses on the early detection and treatment of sleep disorders in individuals who apply for treatment for mental disorders. This randomized controlled trial will result in a database including patients with a variety of mental health problems and a variety of co-occurring sleep disorders. This provides us with the unique opportunity to gain insight into the reciprocal relationship between numerous mental and sleep disorders, and the effects of sleep disorder treatment on mental health. In doing this, we aim to increase knowledge and improve care in individuals with mental disorders and comorbid sleep disorders.

## Supplementary information

**Additional file 1.** Description of questionnaires used in the sleep disorder diagnosis.

## Data Availability

The datasets generated and analyzed during the current study are not publicly available due to confidentiality of the study subjects, but are available from the corresponding author on reasonable request.

## Abbreviations

ADD: Attention Deficit Disorder
ADHD: Attention Deficit Hyperactivity Disorder
ASRM: Altman Self-Rating Mania Scale
ASRS: Adult ADHD Self-Report Scale
ASD: Autism Spectrum Disorder
AQ-50: Autism spectrum Quotient
CBTI: Cognitive Behavioral Therapy -Insomnia
CPAP: Continuous Positive Airway Pressure
CRSD: Circadian Rhythm Sleep disorder
DLMO: Dim Light Melatonin Onset
DSM-5: Diagnostic and Statistical Manual of Mental disorders, *fifth edition*
ESS: Epworth Sleepiness Scale
HSDQ: Holland Sleep disorders Questionnaire
ICSD: International Classification of Sleep disorders
IRLS: International RLS study group rating scale
I-ROC: Individual Recovery Outcome Counter
IRT: Imagination and Rescripting/Rehearsal Therapy
ISI: Insomnia Severity Index
MANSA: Manchester Short Assessment of Quality of Life
MSLT: Multiple Sleep Latency Test
OSA(S): Obstructive Sleep Apnea (Syndrome)
OQ-45: Outcome Questionnaire
PANSS: Positive and Negative Syndrome Scale
PCL-5: Post-traumatic stress disorder Checklist
PSQI: Pittsburgh Sleep Quality Index
PTSD: Posttraumatic Stress Disorder
QIDS- SR: Inventory of Depressive Symptomatology – Self-report
RLS: Restless Legs Syndrome
ROM: Routine Outcome Measurement
SI: Sleep disorder-specific Intervention
SIR: Standardized Incidence Ratio
SRBD: Sleep Related Breathing Disorder
SSD: Schizophrenia Spectrum Disorder
STAI- DY: Spielberger ‘State-Trait Anxiety Inventory – Dutch Y’
TAU: Treatment As Usual
