# Supplementary material for: The clinical relevance of early identification and treatment of sleep disorders in mental health care: protocol of a randomized control trial
Source: BMC Psychiatry. 2020 Jun 24;20:331. doi: 10.1186/s12888-020-02737-3 (PMC7313112; doi:10.1186/s12888-020-02737-3)
Supplement: Supplementary file 1 — Additional file 1. Description of questionnaires used in the sleep disorder diagnosis. [file 12888_2020_2737_MOESM1_ESM.docx]

**Additional file 1**

**Sleep-oriented questionnaires**

*Pittsburgh Sleep Quality Index* (PSQI)
The PSQI [1] is a self-report questionnaire, used to assess subjective sleep quality and disturbances over a 1-month period. Nineteen items produce a global sleep quality score and 7 component scores: sleep quality, sleep latency, sleep duration, habitual sleep efficiency, sleep disturbance, use of sleep medications, and daytime dysfunction. The items use different response categories, including usual bed time, usual wake time, the number of actual hours slept, the number of minutes to fall asleep, 4-point Likert scales pertaining to problem frequency (0= “not during the past month”; 1= “less than once a week”; 2= “once or twice a week”; and 3= “three or more times a week”) and one 4-point Likert scale rating overall sleep quality (0= “very good”; 1= “fairly good”; 2= “fairly bad”; and 3= “very bad”). The PSQI is scored by assigning ordinal values to the items, in order to generate seven equally-weighted component scores, ranging from 0-3. The seven component scales are then summed to yield a global PSQI score, ranging from 0-21. A higher global scores indicates poorer sleep quality, with a cut-off score of 5 distinguishing good sleepers from poor sleepers. The PSQI is a reliable and valid test, with good internal consistency (Chronbach’s alphas ranged from .70 to .83 in most studies) (reviewed in [2])

*Insomnia Severity Index* (ISI)
The ISI [3] is a brief self-report questionnaire measuring the patients’ perception of their insomnia over the past 2 weeks. The ISI consists of 7 items assessing the severity of sleep-onset, sleep maintenance, early morning awakening problems, satisfaction with the current sleep pattern, interference with daily functioning, noticeability of impairment attributed to the sleep problem, and level of distress caused by the sleep problem. Participants are asked to rate the items on a 5-point Likert scale ranging from 0 (= “not at all”) to 4 (= “extremely”). The total score ranges from 0 to 28, with a higher score indicating greater insomnia severity. The ISI is a reliable measure for the assessment of insomnia severity in a clinical population, with sufficient internal consistency (α= .74) and sensitivity to detect changes in the patients’ perception of treatment outcome.

*Morningness - Eveningness Questionnaire* (MEQ)
The MEQ [4] is a 19-item self-report scale in which participants are asked to indicate their habits and life rhythms concerning sleep and waking up, in order to supply information about their most suitable rhythm. Each item consists of 4 or 5 answer choices, corresponding to a definite morning type, moderate morning type, moderate evening type, and definite evening type. A high score indicates definite morningness and a low score indicates definite eveningness for each item. The MEQ is a validated questionnaire with sufficient high internal consistency (α= .82) [5].

*Epworth Sleepiness Scale* (ESS)
The ESS [6] is a measurement of the participants’ general level of daytime sleepiness. Participants are asked to rate how likely they would be to doze off or fall asleep in eight situations, based on their usual way of life in recent times. Answers are given on a scale of 0-3, where 0= “would never doze off”, 1= “slight change of dozing off”, 2= “moderate chance of dozing off” and 3= “high chance of dozing off”. The total score is the sum of the item-scores and ranges between 0 and 24. A higher score indicates a higher level of daytime sleepiness. The ESS is a validated questionnaire with adequate internal consistency (α ranging from .7 to .9) (reviewed in [7]).

*International Restless Legs Syndrome Study Group Rating Scale* (IRLS)
The IRLS [8] is a 10-item scale used to measure disease severity in participants with restless legs syndrome (RLS). The scale provides a subjective assessment of primary (diagnostic) features, intensity and frequency of the disorder and associated sleep problems. Furthermore, questions probing the impact of symptoms on the patients’ mood and daily functioning are included. Each question has a set of five response options, including 0 (= “none”), 1 (= “mild”), 2 (= “moderate”), 3 (= “severe”) and 4 (= “very severe”). The IRLS has been validated, with high levels of internal consistency (α = .94)

**References**

1. Buysse DJ, Reynolds III CF, Monk TH, Berman SR, Kupfer DJ. The Pittsburgh Sleep Quality Index: a new instrument for psychiatric practice and research. Psychiatry research. 1989;28(2):193-213.
2. Mollayeva T, Thurairajah P, Burton K, Mollayeva S, Shapiro CM, Colantonio A. The Pittsburgh sleep quality index as a screening tool for sleep dysfunction in clinical and non-clinical samples: A systematic review and meta-analysis. Sleep medicine reviews. 2016;25:52-73.
3. Bastien CH, Vallières A, Morin CM. Validation of the Insomnia Severity Index as an outcome measure for insomnia research. Sleep medicine. 2001;2(4):297-307.
4. Horne JA, Östberg O. A self-assessment questionnaire to determine morningness-eveningness in human circadian rhythms. International journal of chronobiology 1976;4:97-110.
5. Smith CS, Reilly C, Midkiff K. Evaluation of three circadian rhythm questionnaires with suggestions for an improved measure of morningness. Journal of Applied psychology. 1989;74(5):728.
6. Johns MW. A new method for measuring daytime sleepiness: the Epworth sleepiness scale. Sleep. 1991;14(6):540-5.
7. Kendzerska TB, Smith PM, Brignardello-Petersen R, Leung RS, Tomlinson GA. Evaluation of the measurement properties of the Epworth sleepiness scale: a systematic review. Sleep medicine reviews. 2014;18(4):321-31.
8. International Restless Legs Syndrome Study Group. Validation of the International Restless Legs Syndrome Study Group rating scale for restless legs syndrome. Sleep medicine. 2003;4(2):121-32.
